# Calnexin, an ER-induced protein, is a prognostic marker and potential therapeutic target in colorectal cancer

**DOI:** 10.1186/s12967-016-0948-z

**Published:** 2016-07-01

**Authors:** Deborah Ryan, Steven Carberry, Áine C. Murphy, Andreas U. Lindner, Joanna Fay, Suzanne Hector, Niamh McCawley, Orna Bacon, Caoimhin G. Concannon, Elaine W. Kay, Deborah A. McNamara, Jochen H. M. Prehn

**Affiliations:** Department of Physiology and Medical Physics, Centre for Systems Medicine, Royal College of Surgeons in Ireland, 123 St Stephen’s Green, Dublin 2, Ireland; Department of Colorectal Surgery, Beaumont Hospital, Dublin 9, Ireland; Department of Pathology, Beaumont Hospital and Royal College of Surgeons in Ireland, Dublin 9, Ireland

**Keywords:** Colorectal cancer, ER Stress, Calnexin, GRP78, GRP94, UPR (unfolded protein response)

## Abstract

**Background:**

Colorectal cancer (CRC) is a leading cause of cancer mortality in the Western world and commonly treated with genotoxic chemotherapy. Stress in the endoplasmic reticulum (ER) was implicated to contribute to chemotherapeutic resistance. Hence, ER stress related protein may be of prognostic or therapeutic significance.

**Methods:**

The expression levels of ER stress proteins calnexin, calreticulin, GRP78 and GRP94 were determined in n = 23 Stage II and III colon cancer fresh frozen tumour and matched normal tissue samples. Data were validated in a cohort of n = 11 rectal cancer patients treated with radiochemotherapy in the neoadjuvant setting. The *calnexin* gene was silenced using siRNA in HCT116 cells.

**Results:**

There were no increased levels of ER stress proteins in tumour compared to matched normal tissue samples in Stage II or III CRC. However, increased calnexin protein levels were predictive of poor clinical outcome in the patient cohort. Data were validated in the rectal cancer cohort treated in the neoadjuvant setting. *Calnexin* gene-silencing significantly reduced cell survival and increased cancer cell susceptibility to 5FU chemotherapy.

**Conclusion:**

Increased tumour protein levels of calnexin may be of prognostic significance in CRC, and calnexin may represent a potential target for future therapies.

**Electronic supplementary material:**

The online version of this article (doi:10.1186/s12967-016-0948-z) contains supplementary material, which is available to authorized users.

## Background

Colorectal cancer is one of the most common cancers among men and women in the Western world and the third leading cause of cancer-related mortality. Current treatment options for patients are dependent on disease stage at diagnosis but include surgery and adjuvant chemotherapy [1]. The most common chemotherapy regime currently used in the treatment of Stage II and III colorectal cancer is 5–fluorouracil (5FU), often in combination with oxaliplatin or irinotecan depending on a patients’ clinical status [2, 3]. For patients with Stage II node negative disease, it is unclear if adjuvant therapy will be beneficial, as the majority of these patients will respond to surgery alone, with only a small subset of patients deriving benefit from adjuvant chemotherapy [4]. Adjuvant 5FU-based chemotherapy in stage III disease benefits *c.* 15–20 % of patients, yet the majority of patients will relapse or develop distant metastases within 5 years following surgery [5]. It is therefore important that we improve our ability to identify patients who will benefit from adjuvant chemotherapies and to discover novel drug targets for those patients resistant to current chemotherapeutics.

The endoplasmic reticulum (ER) is an essential cellular organelle responsible for the synthesis; maturation and trafficking of a wide range of proteins and is a critical site for calcium homeostasis. Due to its central role in protein folding and quality control, the ability of the ER to adapt to adverse conditions is essential to the survival of a cell [6, 7]. ER stress is triggered by the accumulation of unfolded or misfolded proteins in the ER and can result from an increased demand for protein synthesis, accumulation of mutant proteins, or from a pathophysiological interference with regular ER functions such as maintenance of Ca^2+^ homeostasis, hypoxia/ischemia or oxidative stress [8]. ER stress triggers the cell’s unfolded protein response (UPR), which alters transcriptional and translational programmes within the cell. The goal of the UPR is to protect the cell from ER stress by decreasing the number of proteins translocated into the ER lumen, by increasing retrotranslocation and augmenting the protein folding capacity of the ER [7]. ER stress induces a number of different proteins such as chaperones from the heat shock protein family including GRP78 and GRP94; Ca^2+^ binding chaperone lectins such as calnexin and calreticulin; and substrate specific chaperones [7].

Somatic gene mutations as well as ischemic conditions may trigger ER stress and elicit the UPR in tumour cells. Rapid growth of tumours can lead to inadequate vascularisation resulting in decreased levels of oxygen, nutrients and glucose as well as altering intracellular calcium levels. In highly oncogenic tumours, protein overproduction or mutations found within tumour cells may cause mutant protein accumulation leading to ER stress [6, 9]. ER stress proteins have been shown to be upregulated in a number of tumour types including breast, lung, liver and prostate tumours and it has been suggested that these altered levels of ER stress proteins are essential for the survival of cancer cells in the inhospitable tumour microenvironment [7, 10, 11]. Thus, in this study we examined whether the ER stress proteins GRP78, GRP94, calnexin and calreticulin are differentially expressed in colorectal tumours compared to matched normal tissue and determined if there was any association between the expression of ER stress proteins and disease stage or clinical outcome in our CRC patient cohort.

## Methods

### Patients

Patient tissue samples were collected and stored in the APOCOLON colorectal tissue biobank at Beaumont Hospital (Dublin, Ireland). Informed consent was received from all patients and ethical approval for use of the stored material was granted by Beaumont Hospital Ethics (Medical Research; 08/62, 11/09 and 16/20) Committee. Snap-frozen colorectal tumour and matched normal tissue from surgical resections of 23 Stage II (n = 8) and III (n = 15) colorectal cancer patients were prospectively collected. Fourteen of the stage III patients and one stage II patient received adjuvant chemotherapy with 10 receiving 5FU and leucovorin, 3 receiving 5FU, leucovorin and oxaliplatin while 1 patient received 5FU, leucovorin and irinotecan, and 1 patient received 5FU, leucovorin, irinotecan and bevacizumab. In 15 cases, patients had a good outcome which was defined as no mortality or disease recurrence within the 4-year follow-up period while 8 cases had a poor outcome, specifically, disease recurrence or death from disease within that timeframe.

Clinical follow-up was obtained for all patients and patient characteristics are summarised in Table 1.

**Table 1:** Details of the disease stage, chemotherapy treatment and disease outcome of the 23 patients within the study

Patient	Stage	Received chemotherapy	Outcome
1	II	*None*	Good
2	II	*None*	Good
3	II	*None*	Poor
4	II	*None*	Good
5	II	*None*	Good
6	II	*None*	Poor
7	II	*None*	Poor
8	II	5FU + Leu	Good
9	III	*None*	Good
10	III	Fol + 5FU + Oxal + Irin	Poor
11	III	5FU + Leu	Good
12	III	5FU + Leu	Good
13	III	5FU + Leu	Poor
14	III	5FU + Leu	Good
15	III	Fol + 5FU + Oxal	Good
16	III	Fol + 5FU + Oxal	Poor
17	III	5FU + Leu	Good
18	III	Fol + 5FU + Oxal	Poor
19	III	5FU + Leu	Good
20	III	5FU + Leu	Good
21	III	5FU + Leu + Irin + bevacizumab	Poor
22	III	5FU + Leu	Good
23	III	5FU + Leu	Good

*5FU* 5-fluorouracil; *Fol* Fluorouracil; *Irin* irinotecan; *Leu* leucovorin; *Oxal* oxaliplatin; *None* no chemotherapy received

### Validation cohort (neoadjuvant setting)

Pre-treatment biopsies were also obtained from a separate cohort of rectal cancer patients by colonoscopy or rigid sigmoidoscopy and stored from the Departments of Surgery, and Pathology, Beaumont Hospital, Dublin, Ireland. Following treatment, surgical resections of these patients was obtained. Ethical approval was obtained by the Beaumont Hospital Ethics (Medical Research; 08/62, 11/09 and 16/20) Committee, and informed consent was obtained from all patients. Resections were evaluated to ensure consistent quality and tumour presence by an experienced pathologist. Clinical follow-up and response to neoadjuvant radio chemotherapy was obtained for all patients and patient characteristics are summarised in Table 2. After neoadjuvant chemo-radiotherapy, patients’ response to therapy was determined using RCPath criteria. Good responders were identified as patients showing complete tumour progression (RCPathA). Poor responders were identified as patients showing partial or no marked tumour regression (RCPathB and C, respectively).

**Table 2:** Details of the disease stage, chemotherapy treatment and disease outcome of the 11 rectal cancer patients of our validation cohort

Patient	Stage	Neo-adjuvant therpay	RCPATH	Outcome
1	III	*50.4/28* + *5FU*	A	Good
2	III	*54.0/30* + *5FU*	A	Good
3	III	*50.4/28* + *5FU*	A	Good
4	II	*50.4/28* + *5FU*	A	Good
5	III	*50.4/28* + *5FU*	B	Poor
6	III	*50.4/28* + *5FU*	B	Poor
7	III	*50.4/28* + *5FU*	B	Poor
8	III	*50.4/28* + *5FU*	B	Poor
9	III	*50.4/28* + *5FU*	C	Poor
10	III	*50.4/28* + *5FU*	C	Poor
11	II	*50.4/28* + *5FU*	C	Poor

*5FU* 5-fluorouracil; 50.4/28, total amount of 50.4 Gy radiotherapy in 28 fractions

### Western blotting

For clinical samples, tissue was lysed in ice-cold tissue lysis buffer [50 mmol/L HEPES (pH 7.5), 150 mmol/L NaCl, 5 mmol/L Na-EDTA] and protease inhibitor cocktail (Calbiochem, Hampshire UK) followed by mechanical homogenization on ice. Following centrifugation (14,000×*g* for 10 min), supernatant was collected and stored at −80 °C until further use. For the cell line experiments, cells were trypsinised and cell pellets collected for each experiment. Cell pellets were then lysed with SDS lysis buffer (2 % SDS, 10 % glycerol, 67.5 mM Tris/Cl^−^ pH 6.8) and protease inhibitor cocktail. The samples were then heated to 95 °C while shaking (600 rpm for 20 min) following which the supernatant was collected and stored at −80 °C until further use. Protein concentration was measured using micro BCA (bicinchoninic acid) assay (Pierce, Rockford, IL). Total protein (20 µg) was resolved using SDS–PAGE, transferred to nitrocellulose membranes and blocked in TBS-T/5 % milk for 1 h. Membranes were incubated in primary antibody, calnexin (1:1000, rabbit polyclonal), calreticulin (1:1000, mouse monoclonal) and KDEL (1:500, mouse monoclonal) (Enzo Life Sciences, Exeter, UK) overnight at 4 °C, washed in TBS-T and incubated in the appropriate horseradish peroxidase secondary antibody at room temperature for 1 h. The KDEL antibody used binds the amino acid sequence Lys-Asp-Glu-Leu (KDEL) present at the carboxy-terminus of GRP78 and GRP94 thus this antibody detects both GRP78 and GRP94. Detection of protein bands was carried out using chemiluminescence (EMD Millipore, Billerica, MA, USA) on a LAS-3000 Imager (FUJIFILM UK Ltd. Systems, Bedford UK). Densitometry on each band was carried out using ImageJ software and normalized to Actin (1:5000 mouse monoclonal, Sigma Aldrich, Dublin, Ireland) loading control. Actin normalized intensities were then used to determine tumor to matched normal ratios for each patient.

### Calnexin gene silencing in HCT116 cells

HCT116 colon cancer cells were transfected with calnexin siRNA and control scramble siRNA (Santa Cruz Biotechnology, Dallas, TX, USA). The cells were seeded on 6-well tissue culture plates (2 × 10^5^ cells/well) and transfected after 24 h. After a further 24 h, cells were harvested to confirm knockdown of calnexin expression by western blot analysis.

### Flow cytometry

Following transfection of HCT116 cells with *calnexin* or control scramble siRNA for 24 h and subsequent treatment with 5FU or vehicle for 48 h, cells were harvested and the induction of cell death was assessed by Annexin V and PI staining using flow cytometry. Cells were incubated at room temperature in binding buffer (10 mM HEPES, 135 mM NaCl, 5 mM CaCl_2_) which contained an Annexin V-FITC conjugate (1 μl/ml; BioVision, Mountain View, CA, USA) and propidium iodide (PI; 1 μl/ml, BioVision) for 15 min. Cells were counted in a Cyflow ML 16 flow cytometer (Partec, Münster, Germany). Excitation of Annexin V-FITC was done with a 488 nm laser and fluorescence emission was collected in the FL1 channel through a 520 nm band pass filter. PI was excited with a 488 nm laser and fluorescence emission was collected in the FL3 channel through a 620 nm long pass filter. 1 × 10^4^ gated cells were acquired for each sample and analyzed using the Flowmax software (Partec).

### Clonogenic survival assay

HCT116 cells were transfected with calnexin or control scramble siRNA for 24 h and then plated for a clonogenic survival assay. Transfected cells were seeded on a 6-well plate (1 × 10^3^ cells/well) in medium (3 ml) and incubated at 37 °C for 5 days. After 5 days, the medium was replaced with fresh medium and cells were incubated for a further 9 days at which time medium was removed and clonogenic reagent (2 ml; 50 % ethanol, 0.25 % 1, 9 dimethlyene blue) was added to each well and incubated at room temperature for 45 min. The cells were then washed with PBS and the number of colonies was counted.

### Immunohistochemistry

Tissue sections were cut at 3 μm on to Leica Microsystems Plus slides and were baked over night at 37 °C prior to immunostaining. All staining was performed on a Leica Bond III automated immunostainer from Leica Biosystems, Newcastle, UK. Sections were loaded onto the system and the relevant programme was selected. The Bond-III system dewaxed slides and then carried out a 20 min pre-treatment with BOND Epitope Retrieval Solution I (Cat. no. AR9961). Antibodies were chosen based on their suitability for use on FFPE tissue and optimised. Primary antibodies were diluted in Bond Primary Antibody Diluent (Cat. no. AR9352)—1:400 diluted rabbit polyclonal anti-Calnexin (ADI-SPA-860, Enzo Life Sciences, Exeter, UK) and were added to the sections for 20 min. Detection and visualisation of stained cells was achieved using the Bond Polymer Refine Detection Kit (Cat. no. DS9800), using diaminobenzidine tetrachloride (DAB) as the chromagen. Tissues were counterstained with haematoxylin and were cover slipped. Negative controls for rabbit antibody (Negative Control Rabbit Immunoglobulin fraction of serum from non-immunized rabbits, IS600, Dako) were included and no staining was observed in these controls.

### Statistical analysis

All results from the patient samples and cell line work were analysed using GraphPad InStat software. The expression of individual proteins in tumour and matched normal samples was examined with two-sided Wilcoxon signed-rank test for related variables. The expression of the tumour to normal ratios was calculated using Mann–Whitney U test for independent samples. In vitro data were analysed by basic *t* tests and one-way ANOVA with Student–Newman–Keuls post hoc test where appropriate. Results were considered significant when the p-value was <0.05.

## Results

### Expression of ER Stress proteins in stage II and stage III colorectal cancer patient samples

**Fig. 1:**
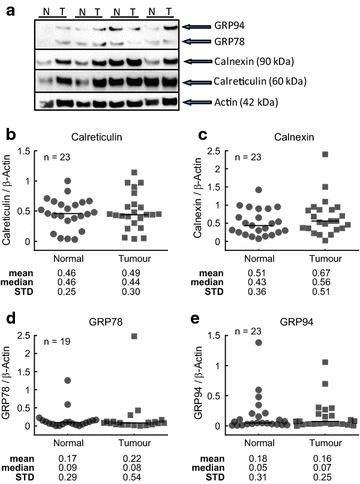
Levels of ER stress proteins in a cohort of stage II and stage III CRC patients. Sample western blots showing the levels of the ER stress proteins GRP94, GRP78, calnexin and calreticulin in colorectal tumour (T) and matched normal (N) colonic tissue (**a**). *Scatter plots* depicting expression of calreticulin (**b**), calnexin (**c**), GRP78 (**d**) and GRP94 (**e**) in colorectal tumour and matched normal tissue. Differences between tumour and matched normal tissue were assessed by Wilcoxon signed rank test. The mean, median and standard deviation *STD* were stated below the* panels*

The activation of the UPR in response to ER stress has the potential to allow cancer cells to survive adverse conditions that may otherwise lead to apoptosis. In order to examine the potential role of ER stress in colorectal cancer we determined the expression of the ER stress proteins calreticulin (Fig. 1b), calnexin (Fig. 1c), GRP78 (Fig. 1d) and GRP94 (Fig. 1e) in colorectal tumour and matched normal tissue from patients with Stage II (n = 8) and Stage III (n = 15) disease (Additional file 1: Table S1). Sample images of the Western blots of the ER stress proteins can be seen in Fig. 1a. A two-sided Wilcoxon signed rank test analysis of the expression of the ER stress proteins in the tumour samples and matched normal tissue established that there was no differential expression of the proteins between tumour and normal tissue (Fig. 1b, c, d, e). Furthermore, the levels of GRP78 and GRP94 were very low across the total patient cohort (Fig. 1d, e respectively). Additional file 2: Figure S1 provides an additional analysis with paired samples highlighted.

### Expression of ER stress proteins as a predictor of disease stage

**Fig. 2:**
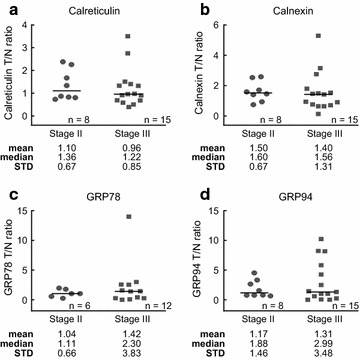
Levels of ER stress proteins in CRC tissue do not correlate with disease stage. Scatter plots showing the tumour/normal ratio of the ER stress proteins calreticulin (**a**), calnexin (**b**), GRP78 (**c**) and GRP94 (**d**) in stage II versus stage III CRC patient tissue. Differences between tumour/normal ratios of the ER stress protein in stage II and stage III CRC were assessed by Mann–Whitney U test. The mean, median and STD were stated below the* panels*

Since there was no differential expression of ER stress proteins between tumour and normal tissue in our CRC patient samples, we next assessed if the ratio of ER stress protein expression in tumour compared to normal tissue could be predictive of disease stage. We preferred to perform a tumour/normal ratio analysis to better control for potential differences in sample acquisition times and sample preparations among individually collected patient samples. Patient samples were subdivided by disease stage, stage II and stage III, and the ratio of calreticulin (Fig. 2a), calnexin (Fig. 2b), GRP78 (Fig. 2c) and GRP94 (Fig. 2d) in tumour versus normal tissue was evaluated. This analysis determined that there was no statistically significant difference between the ratios of ER stress protein expression in tumour versus normal tissue in samples from patients with Stage II CRC compared with samples from patients with Stage III CRC. Thus, the tumour/normal ratios of ER stress proteins are not predictive of disease stage. We likewise found no differences when comparing tumour and matched normal samples independently (Additional file 3: Figure S2).

### Expression of ER stress proteins as predictors of clinical outcome

**Fig. 3:**
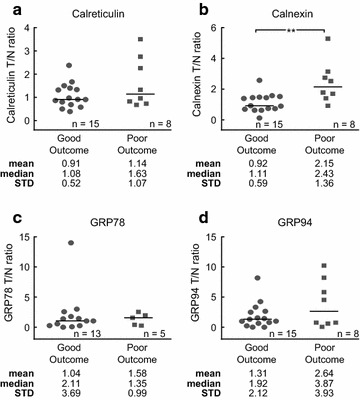
Levels of ER stress proteins correlate with poor clinical outcome. *Scatter plots* of tumour/normal ratios of the ER stress proteins calreticulin (**a**), calnexin (**b**), GRP78 (**c**) and GRP94 (**d**) in the total CRC patient cohort with good versus poor outcomes. The tumour/normal ratio of calnexin was significantly increased in those patients had a poor clinical outcome compared with patients with good clinical outcome (**p < 0.01; Mann–Whitney U Test). The mean, median and STD were stated below the* panels*

Since ER stress may alter tumour growth and chemosensitivity of tumour cells, it may also influence clinical outcomes and patient survival. Therefore, we next examined whether the expression of ER stress associated proteins in tumour tissue compared to normal tissue was associated with clinical outcome. Patient samples were divided based on the clinical outcome of the patients with good outcome being defined as no mortality or disease recurrence within the 4 year follow-up period while poor outcome was defined as disease recurrence or death from disease within that timeframe. The tumour/normal ratios of calnexin (p = 0.0055; Fig. 3b) were significantly increased in samples from patients who had poor clinical outcome. No significant differences in the tumour/normal ratios of calreticulin (Fig. 3a), GRP78 (Fig. 3c) and GRP94 (Fig. 3d) were detected. We likewise found elevated protein levels of calnexin in tumour (p = 0.0030) but not matched normal tissue of patients with poor outcome when comparing tumour and matched normal samples independently (Additional file 4: Figure S3). In a separate analysis, we analysed only those patients who received 5FU based adjuvant chemotherapy. The tumour/normal ratios of calnexin (p = 0.0093; Additional file 5: Figure S4B) were significantly increased in samples from patients who received chemotherapy and had poor clinical outcome. No significant differences in the expression of calreticulin, GRP78 and GRP94 in tumour compared to matched normal tissue were detected in samples from patients with good or poor outcome who received chemotherapy (Additional file 5: Figure S4A, C, D).

**Fig. 4:**
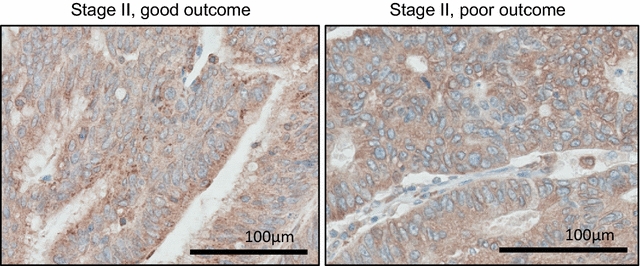
Immunohistochemical analysis of calnexin expression in CRC. Immunohistochemical staining of paraffin-embedded human colorectal cancer patient tissue using anti-calnexin polyclonal antibody. Tissue section on the *left* represents one CRC stage II good responder and tissue section on the *right* shows a CRC stage II poor responder. Calnexin immunostaining localised to the cytoplasm in both good and poor responsers. Images are representative of the n = 15 and n = 8 good and poor responders analysed by immunohistochemistry. Hematoxylin staining was performed to visualise nuclei (*blue* *colour*). *Scale* *bar* corresponds to 100 µm

We also explored whether there were potential differences in the subcellular localization of calnexin in patients with good or poor outcome, and performed an immunohistochemical analysis of in good and in poor outcome cases. However, we observed little variation in the subcellular localization of calnexin in good and poor responders, with cytoplasmic, punctuate calnexin staining in all tumor cells (Fig. 4).

### Calnexin is a prognostic biomarker in rectal cancer patients treated in the neoadjuvant setting

To validate our findings, pre-treatment biopsy tumour tissue samples were obtained at colonoscopy or rigid sigmoidoscopy (Table 1) from 11 rectal cancer patients. Using quantitative Western blotting, the expression levels of calnexin in tumour biopsy tissue were determined in all 11 patients (Additional file 1: Table S2). After neoadjuvant chemoradiotherapy (50.4/28 + 5FU), patients’ response to therapy was determined on surgical resected tissue. Patients were either classified as good outcome (RCPath A; complete tumour regression) or bas outcome [RCPath B (partial tumour regression) or RCPath C (no marked tumour regression)].

**Fig. 5:**
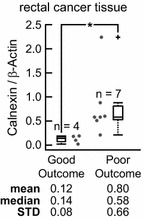
Calnexin levels correlate with poor clinical outcome in rectal cancer patients treated in the neoadjuvant setting. The calnexin level was significantly increased in rectal cancer patients who received neo-adjuvant radiochemotherapy (50.4 Gy + 5FU) and had a poor clinical outcome (RCPath B & C) compared with patients who received neo-adjuvant radiochemotherapy with good clinical outcome (RCPath A; *p = 0.0061; Mann–Whitney U Test; Bonferroni corrected significance level α = 0.0125)

We found statistically significant differences of the Calnexin level between the RCPaths (Kruskal–Wallis test p = 0.0295). After adjustment for multi-comparison (Bonferroni correction, α = 0.0125), Calnexin levels were significant up-regulated in poor responders (RCPath B or C), compared to good responders (RCPath A; Mann–Whitney U test p = 0.0286) (Fig. 5).

### The effect of calnexin knockdown on clonogenic survival and the induction of cell death by 5FU in HCT116 cells

**Fig. 6:**
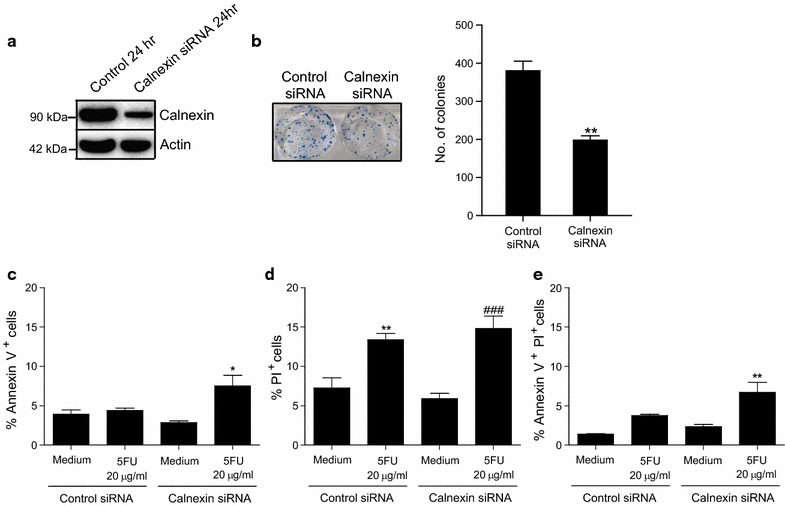
Gene silencing of *calnexin* attenuates HCT116 colon cancer cell survival. Western blot analysis depicting decreased expression of calnexin in HCT116 colon cancer cells following transfection for 24 h with calnexin siRNA compared with control siRNA (**a**). Sample image of a clonogenic survival assay and histogram depicting total colony number following transfection of HCT116 cells with control or calnexin siRNA for 24 h and subsequent colony formation for a further 14 days (**b**; n = 3). Annexin V and PI staining of HCT116 cells transfected with control or calnexin siRNA for 24 h followed by treatment with 5FU (20 µg/ml) for 48 h as determined by flow cytometry (**c**, *p < 0.05 versus HCT116 cells transfected with control siRNA and treated with 5FU; **d**, **p < 0.01 versus HCT116 cells transfected with control siRNA and treated with vehicle, ^###^p < 0.001 versus HCT116 cells transfected with calnexin siRNA and treated with vehicle; E, **p < 0.01 versus HCT116 cells transfected with control siRNA and treated with 5FU; *graphs* representative of three independent experiments)

To determine a role for calnexin in colorectal cancer cell survival and 5FU-induced cell death, we silenced *calnexin* gene expression in HCT116 cells using siRNA-mediated gene silencing. We first ascertained a successful reduction of calnexin protein levels in HCT116 cells 24 h post *calnexin* siRNA transfection as determined by Western blot analysis (Fig. 6a). A clonogenic survival assay was utilised to assess the ability of HCT116 cells to survive and proliferate following *calnexin* gene silencing. Gene silencing of *calnexin* significantly decreased clonogenic survival (p = 0.0021, Fig. 6b).

Since calnexin expression was significantly elevated in patients with poor outcome who received 5FU-based chemotherapy, we next examined the effect of *calnexin* knockdown on chemosensitivity in HCT116 cells. Following transfection with calnexin siRNA for 24 h, HCT116 cells were treated with 5FU (20 µg/ml) for 48 h and the induction of cell death was determined by Annexin V and PI staining using flow cytometry. The percentage of Annexin V^+^ cells, indicating apoptosis, was significantly increased when HCT116 cells transfected with calnexin siRNA and treated with 5FU were compared with HCT116 cells transfected with control siRNA and treated with 5FU [Fig. 6c, *p < 0.05 versus HCT116 cells transfected with control siRNA and treated with 5FU]. Treatment with 5FU significantly increased the percentage of PI^+^ cells in both control and calnexin siRNA transfected HCT116 cells with no significant effect of calnexin knockdown observed [Fig. 6d, **p < 0.01 versus HCT116 cells transfected with control siRNA and treated with vehicle; ^###^p < 0.001 versus HCT116 cells transfected with calnexin siRNA and treated with vehicle]. While treatment of control siRNA transfected HCT116 cells with 5FU did not significantly increase the percentage of Annexin^+^PI^+^ cells, knockdown of calnexin synergised with 5FU treatment to significantly enhance the percentage of HCT116 Annexin^+^PI^+^ cells [Fig. 6e, **p < 0.01 versus HCT116 cells transfected with control siRNA and treated with 5FU].

## Discussion

The significant findings of this study identify the ER calcium binding chaperone calnexin as promising prognostic marker and therapeutic target in colorectal cancer. We have demonstrated that Calnexin levels correlated with clinical response in the total population of stage 2 and 3 CRC patients and in patients who received 5FU-based adjuvant chemotherapy. These findings were validated in rectal cancer patients treated with chemoradiation therapy in the neoadjuvant setting. Furthermore, we establish the importance of calnexin in colorectal cancer cell survival and responsiveness to 5FU-based chemotherapy.

The rapid proliferation of tumour cells requiring increased protein translation coupled with the inadequate blood supply has been suggested to lead to ER stress and activation of the UPR [8]. A tumour cell’s ability to tolerate ER stress may account for its capacity to grow under adverse conditions and to resist current chemotherapeutic regimens. The ER resident chaperone GRP78, which plays a key role in sensing misfolded proteins and activating the UPR, has been shown to be up regulated in a variety of cancer cell lines and human specimens including breast, lung, liver and prostate cancer [7, 12]. However, we demonstrate that the expression of GRP78 was low in both tumour and matched normal tissue in our colorectal cancer patient samples and the expression levels of GRP78 failed to correlate with disease stage or clinical outcome. In contrast to our findings, other studies have demonstrated that the level of GRP78 positively correlated with tumour progression in primary human breast [13], liver [14] and melanoma [15] tumour tissue. Interestingly, a study by Hardy et al. [16] showed that the localisation of GRP78 within colon cancer cells was crucial to their tumourigenicity with cells expressing GRP78 in the ER but not on the cell surface being highly proliferative and capable of inducing liver metastasis in a human metastatic colorectal carcinoma model in mice [16]. While we did not detect differences in the subcellular localisation of calnexin by immunohistochemistry among good and poor responders, it is possible that the localisation of GRP78 and not its overall expression level or tumour/normal ratio determines its contribution to disease stage or clinical outcome of the patient. This was not further analysed in this study as we were not able to identify a solely GRP78 specific antibody compatible with FFPE samples.

We also demonstrate that the glucose regulated ER resident molecular chaperone GRP94 is lowly expressed in our colorectal cancer tumour and matched normal samples. Previous studies indicate that overexpression of GRP94 associated with cellular transformation and tumourigenicity in cancer cell lines [17]. In contrast, GRP94 is known to elicit anti-tumour immune responses in mouse models [18, 19] through its ability to bind and present tumour specific antigens [20] and induce maturation of dendritic cells [21]. However, we found no evidence that the expression of GRP94 correlated with disease stage or clinical outcome in our cohort of CRC patients.

Calcium, the universal signaling molecule involved in processes such as protein modification, protein folding and cell survival and death signaling, is stored in the ER, where it is buffered by that calcium binding ER chaperones calreticulin and calnexin. Our data demonstrate that an increased tumour/normal ratio of calnexin, but not calreticulin, was significantly associated with poor clinical outcome in patients that received chemotherapy. Calreticulin has been implicated in affecting cell sensitivity to apoptosis, with calreticulin deficiency lowering ER luminal Ca^2+^ concentrations and protecting cells from apoptosis [22] while overexpression of calreticulin sensitizes cells to the induction of apoptosis [22, 23]. Expression of the calcium binding ER chaperone calnexin has been shown to be a good predictor of cell sensitivity to ER stress-mediated cell death [24] and to mediate resistance to apoptosis through its caspase-dependent cleavage [25]. In the present study, we significantly expand on the key role of calnexin in colon cancer cell survival by demonstrating that gene silencing of *calnexin* decreases clonogenic cell survival. Furthermore, we show that gene silencing of *calnexin* in combination with 5FU treatment significantly increases the percentage of cells undergoing both early (Annexin V^+^ only cells) and late apoptosis (Annexin V^+^PI^+^ cells) as well as inducing significant levels of necrosis (PI^−^ only cells and Annexin V^+^PI^+^ cells). It has been previously demonstrated that cells defective in apoptosis undergo autophagy-induced necrotic-like cell death as a result of prolonged ER stress [26], which could explain the induction of necrosis observed in our study.

CRC patients with tumors exhibiting high microsatellite instability (MSI) were previously shown not to benefit from 5FU-based adjuvant chemotherapy [27]. Our finding might suggest that, targeting *calnexin* in combination with 5FU treatment might improve overall survival among patients with high MSI, since HCT-116 cells are known to feature clinical and cytological characteristics of CRC patients stratified for high MSI [28].

## Conclusions

In summary, this study indicates that the calnexin protein levels may represent a new indicator of poor clinical outcome of stage II/III colorectal cancer patients who received 5FU-based chemotherapy. Furthermore, our in vitro studies confirmed the importance of calnexin for colorectal cancer cell growth and proliferation and also demonstrated that *calnexin* deficiency could enhance responsiveness to 5FU-based-chemotherapy. Together, these results suggest a possible role for the calcium binding chaperone calnexin as a prognostic biomarker in stage 2 and 3 colorectal cancer and as a potential therapeutic target, which requires further validation in clinical and pre-clinical studies.

## Additional files

10.1186/s12967-016-0948-z and **S2**. Densitometric levels for ER stress proteins calreticulin, calnexin, GRP78 and GRP94 in CRC patients by quantitive western blotting.
10.1186/s12967-016-0948-z Box and scatter plots depicting expression of calreticulin (A), calnexin (B), GRP78 (C) and GRP94 (D) in colorectal tumour and matched normal tissue. Differences between tumour and matched normal tissue were assessed by Wilcoxon signed rank test. Paired samples with lower levels in tumour compared to matched normal tissue were mapped with a solid line. Paired samples with higher levels in tumour compared to matched normal tissue were mapped with a dotted line.
10.1186/s12967-016-0948-z Box plots showing tumour and matched normal levels of the ER stress proteins calreticulin (A), calnexin (B), GRP78 (C) and GRP94 (D) in stage II versus stage III CRC patient tissue. Differences between expression levels of the ER stress proteins in stage II and stage III CRC were assessed by Mann Whitney U test.
10.1186/s12967-016-0948-z Box plots of tumour and matched normal levels of the ER stress proteins calreticulin (A), calnexin (B), GRP78 (C) and GRP94 (D) in the total CRC patient cohort in good versus poor outcome cases. The expression level of calnexin in tumour tissue was significantly increased in patients with poor clinical outcome compared with patients with good clinical outcome (**p<0.01; Mann Whitney U Test).
10.1186/s12967-016-0948-z Levels of calnexin correlate with poor clinical outcome in patients that received adjuvant chemotherapy. Scatter plots of tumour/normal ratios of the ER stress proteins calreticulin (A), calnexin (B), GRP78 (C) and GRP94 (D) in patients who received 5FU based adjuvant chemotherapy. The tumour/normal ratio of calnexin was significantly increased in those patients who received adjuvant chemotherapy and had a poor clinical outcome compared with patients who received adjuvant chemotherapy with good clinical outcome (**p<0.01; Mann Whitney U Test).

## Abbreviations

5FU: 5–fluorouracil
BCA: bicinchoninic acid
CRC: colorectal cancer
DAB: diaminobenzidine tetrachloride
EDTA: ethylenediaminetetraacetic acid
ER: endoplasmic reticulum
Fol: fluorouracil
GRP78: 78 kDa glucose-regulated protein/binding immunoglobulin protein
GRP94: heat shock protein 90 kDa beta member 1
Irin: irinotecan
h: hours
HEPES: 2-[4-(2-hydroxyethyl)piperazin-1-yl]ethanesulfonic acid
KDEL: amino acid sequence Lys-Asp-Glu-Leu
Leu: leucovorin
M: molar
min: minute
MSI: microsatellite instability
Oxal: oxaliplatin
p: *p* value
PI: propidium iodide
RCPath: standard to describe tumour regression following neoadjuvant therapy published by the Royal College of Pathologists
rpm: revolutions per minute
SDS: sodium dodecyl sulfate
siRNA: small interfering RNA
Suppl.: supplementary
UPR: unfolded protein response
