# Erratum to: Calnexin, an ER stress-induced protein, is a prognostic marker and potential therapeutic target in colorectal cancer

**DOI:** 10.1186/s12967-016-0984-8

**Published:** 2016-07-26

**Authors:** Deborah Ryan, Steven Carberry, Áine C. Murphy, Andreas U. Lindner, Joanna Fay, Suzanne Hector, Niamh McCawley, Orna Bacon, Caoimhin G. Concannon, Elaine W. Kay, Deborah A. McNamara, Jochen H. M. Prehn

**Affiliations:** 1 Department of Physiology and Medical Physics, Centre for Systems Medicine, Royal College of Surgeons in Ireland, 123 St Stephen’s Green, Dublin 2, Ireland; 2 Department of Colorectal Surgery, Beaumont Hospital, Dublin 9, Ireland; 3 Department of Pathology, Beaumont Hospital and Royal College of Surgeons in Ireland, Dublin 9, Ireland

## Erratum to: J Transl Med (2016) 14:196 DOI 10.1186/s12967-016-0948-z

Unfortunately, the original version of this article [1] contained an error. The title was included incorrectly. The correct can be found here. The title has been corrected in the original article and is also included correctly in this erratum.
