# Supplementary figures and images for: Calnexin, an ER-induced protein, is a prognostic marker and potential therapeutic target in colorectal cancer
Source: J Transl Med. 2016 Jul 1;14:196. doi: 10.1186/s12967-016-0948-z (PMC4930591; doi:10.1186/s12967-016-0948-z)

# Supplementary Figure 1

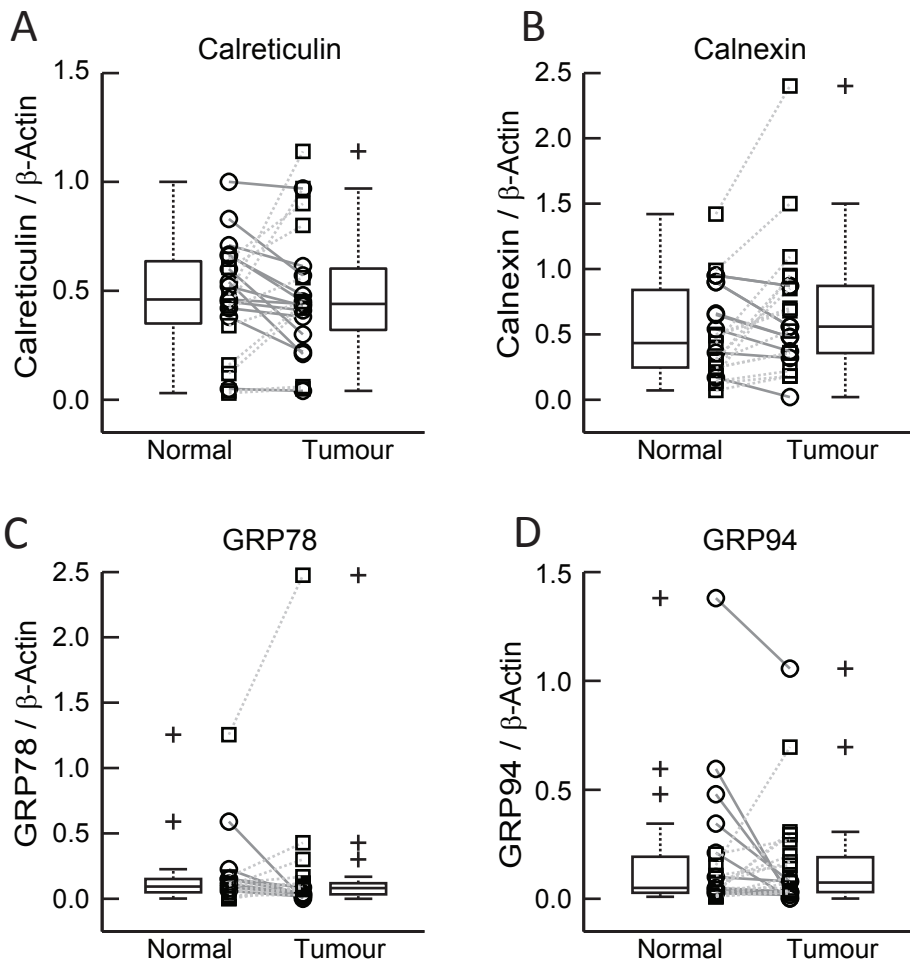

Supplement: Supplementary file 2 — 10.1186/s12967-016-0948-z Box and scatter plots depicting expression of calreticulin (A), calnexin (B), GRP78 (C) and GRP94 (D) in colorectal tumour and matched normal tissue. Differences between tumour and matched normal tissue were assessed by Wilcoxon signed rank test. Paired samples with lower levels in tumour compared to matched normal tissue were mapped with a solid line. Paired samples with higher levels in tumour compared to matched normal tissue were mapped with a dotted line. [file 12967_2016_948_MOESM2_ESM.pdf]

# Supplementary Figure 2

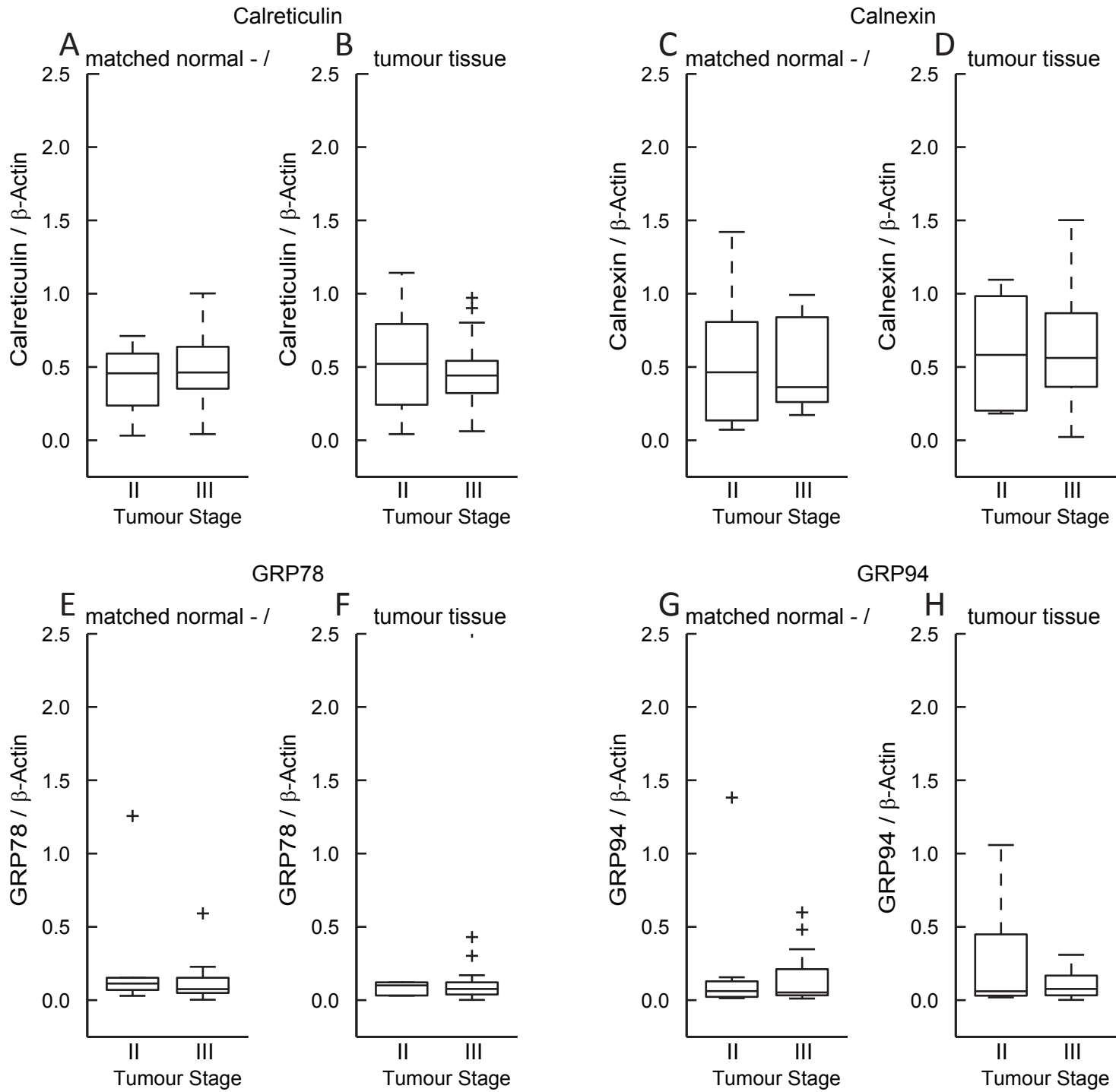

Supplement: Supplementary file 3 — 10.1186/s12967-016-0948-z Box plots showing tumour and matched normal levels of the ER stress proteins calreticulin (A), calnexin (B), GRP78 (C) and GRP94 (D) in stage II versus stage III CRC patient tissue. Differences between expression levels of the ER stress proteins in stage II and stage III CRC were assessed by Mann Whitney U test. [file 12967_2016_948_MOESM3_ESM.pdf]

# Supplementary Figure 3

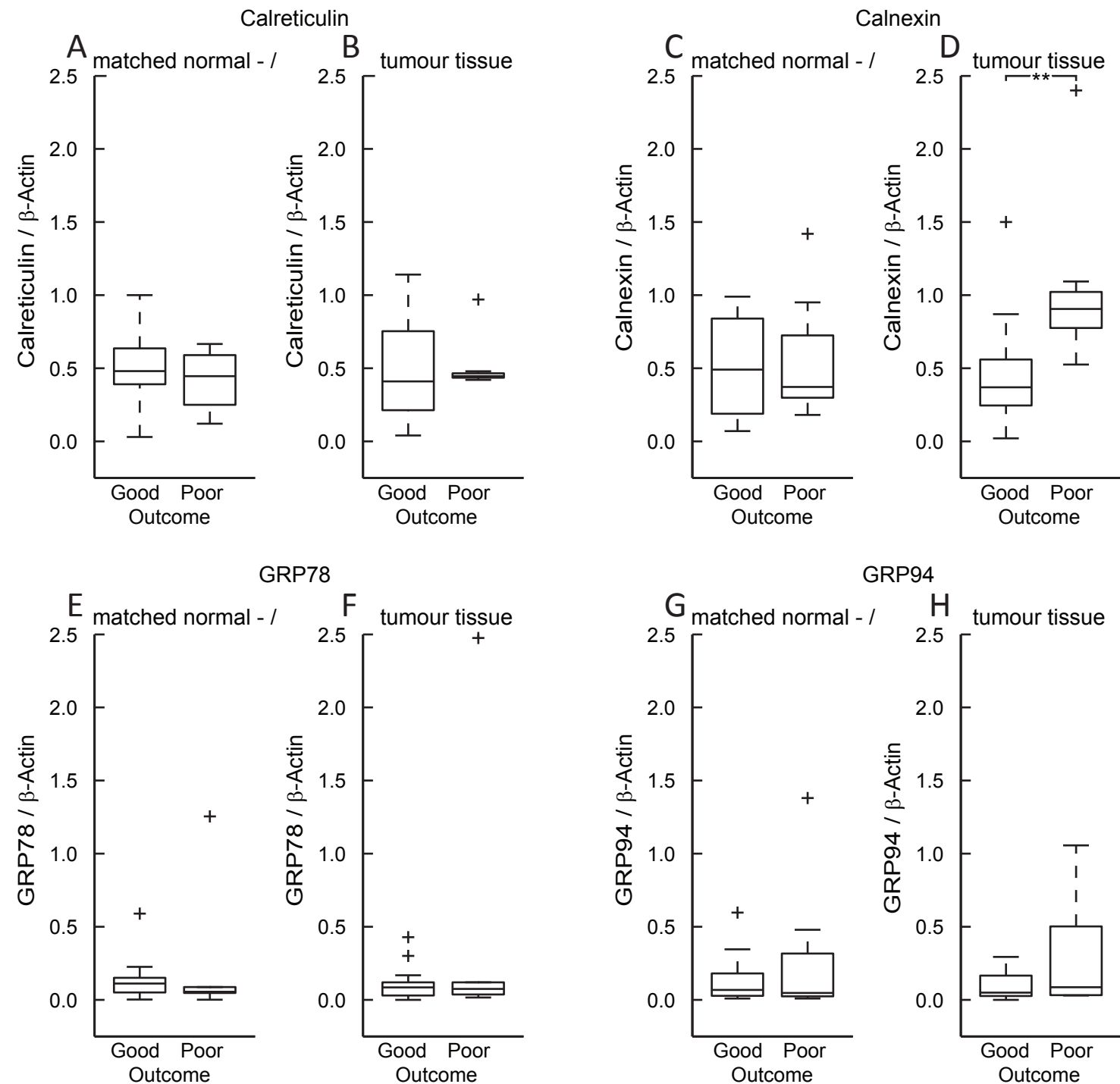

Supplement: Supplementary file 4 — 10.1186/s12967-016-0948-z Box plots of tumour and matched normal levels of the ER stress proteins calreticulin (A), calnexin (B), GRP78 (C) and GRP94 (D) in the total CRC patient cohort in good versus poor outcome cases. The expression level of calnexin in tumour tissue was significantly increased in patients with poor clinical outcome compared with patients with good clinical outcome (**p<0.01; Mann Whitney U Test). [file 12967_2016_948_MOESM4_ESM.pdf]

# Supplementary Figure 4

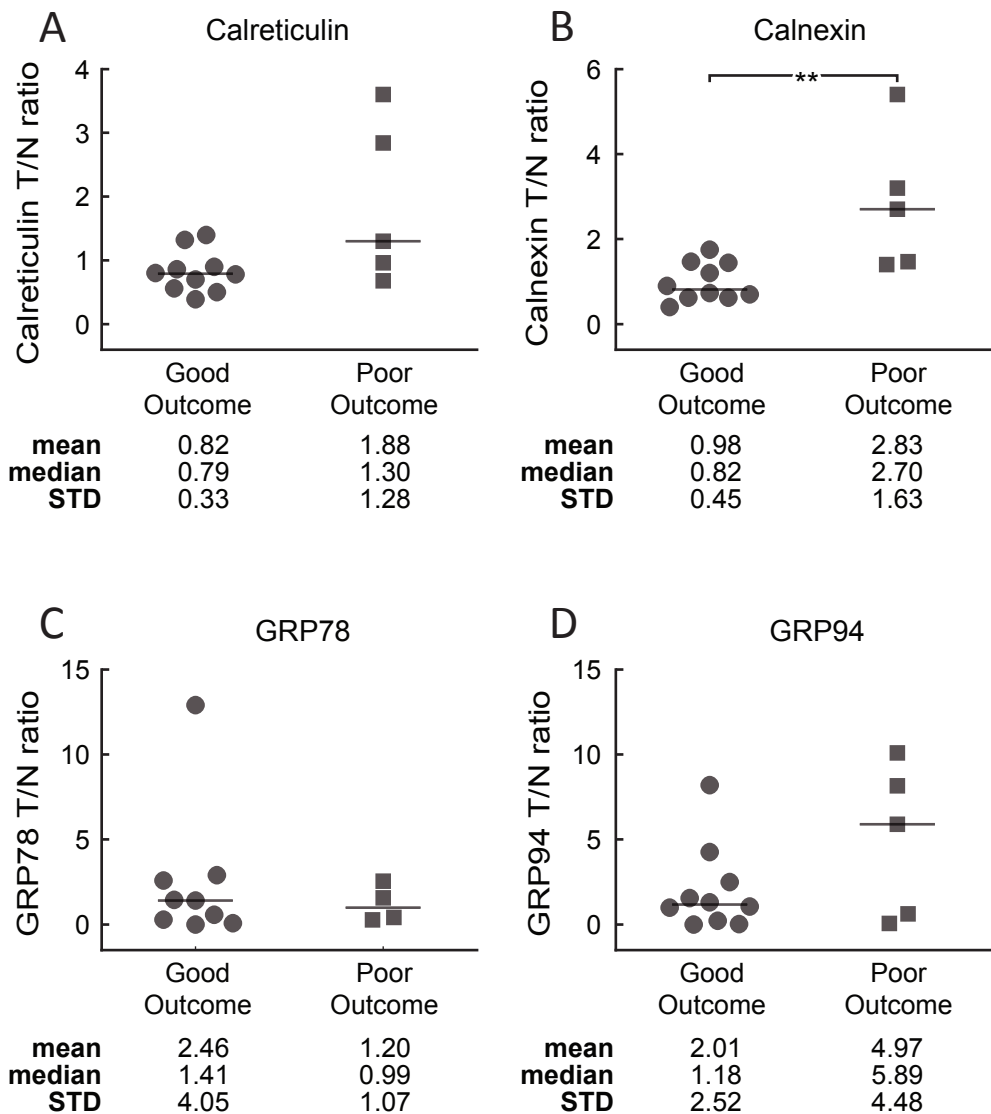

Supplement: Supplementary file 5 — 10.1186/s12967-016-0948-z Levels of calnexin correlate with poor clinical outcome in patients that received adjuvant chemotherapy. Scatter plots of tumour/normal ratios of the ER stress proteins calreticulin (A), calnexin (B), GRP78 (C) and GRP94 (D) in patients who received 5FU based adjuvant chemotherapy. The tumour/normal ratio of calnexin was significantly increased in those patients who received adjuvant chemotherapy and had a poor clinical outcome compared with patients who received adjuvant chemotherapy with good clinical outcome (**p<0.01; Mann Whitney U Test). [file 12967_2016_948_MOESM5_ESM.pdf]
